# Dual‐Locus Metabarcoding Reveals Dry‐Season Dietary Differentiation Between Elephants and Impalas in a South African Savanna

**DOI:** 10.1002/ece3.74487

**Published:** 2026-10-11

**Authors:** Kyle R. van Wyk, Danielle Botha, Sandra Barnard, Frances Siebert

**Affiliations:** ^1^ Unit for Environmental Sciences and Management North‐West University Potchefstroom South Africa

**Keywords:** dietary analysis, dietary niche overlap, forbs, herbivore coexistence, plant functional groups

## Abstract

Large herbivores in African savannas experience intense nutritional constraints during the dry season when declining forage quality intensifies competition for limited resources. Sympatric mixed feeders are therefore expected to differ in their use of forage resources, yet specific plant taxa underlying these dietary differences remain poorly resolved. Here, we used dual‐locus DNA metabarcoding (*rbc*L and *trn*L) to characterise and compare dry‐season diets of African elephants ( *Loxodonta africana* ) and impalas ( *Aepyceros melampus* ) in a semi‐arid savanna ecosystem in South Africa. Despite substantial differences in body size and digestive physiology, dietary alpha diversity did not differ significantly between herbivores for either marker. In contrast, FOO‐based Jaccard beta‐diversity analyses revealed significant differences in dietary composition between species for both markers, with the strongest differentiation detected using *trn*L. Pianka's niche overlap index and relative read abundance analyses supported these patterns, showing lower overlap between species than within‐species. Both herbivores relied heavily on woody taxa during the dry season. Elephants consumed a narrower assemblage dominated by browse genera such as *Grewia*, *Dichrostachys* and *Vachellia*, whereas impalas incorporated a more compositionally diverse mixture of woody, graminoid and forb taxa. Our findings demonstrate that dry‐season dietary differentiation between these mixed feeders is associated less with differences in overall dietary breadth than with fine‐scale taxonomic differences and contrasting use of woody, graminoid and forb resources. These results highlight the potential importance of maintaining taxonomic and growth‐form heterogeneity in forage resources to support savanna herbivore assemblages under increasingly variable environmental conditions.

## Graphical abstract

We applied dual‐locus DNA metabarcoding to compare dry‐season diets of African elephants and impalas in a South African savanna. Despite similar dietary diversity, the two species differed substantially in dietary composition, with differentiation associated with fine‐scale taxonomic differences rather than overall dietary breadth.

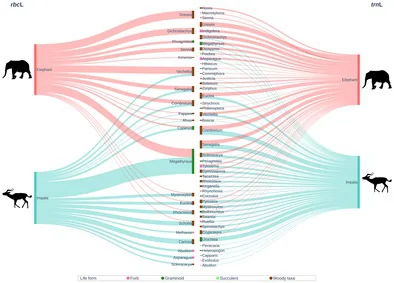

## 1 Introduction

African savannas are characterised by strong seasonal rainfall contrasts that drive pronounced fluctuations in forage availability and quality (Staver et al. 2011; Fynn and Provenza 2023; Nketsang et al. 2025). During the dry season, C_4_ grasses senesce, resulting in sharp increases in fibre and lignin concentrations, reduced digestibility and severe dietary limitations for large mammalian herbivores, culminating in a pronounced dry‐season nutritional bottleneck (Van Soest 1994; Kos et al. 2012; Abraham et al. 2019; Fynn and Provenza 2023). To contend with this recurrent scarcity of grazing resources and decline in nutritional quality, herbivores employ two broad, non‐exclusive strategies: spatial redistribution through migration, or compositional adjustments in diet that permit exploitation of alternative plant resources (Fryxell and Sinclair 1988; Codron et al. 2007; Abraham et al. 2019; Wang et al. 2025). These alternatives typically consist of C_3_ woody plants and perennial forbs, which often retain foliage and relatively high nutrient quality throughout the dry season, potentially serving as important dry‐season forage resources when grass quality declines (Lagendijk et al. 2005; Duncan and Poppi 2008; Abraham et al. 2019; Siebert and Dreber 2019; Pansu et al. 2019). This asymmetry in seasonal resource reliability highlights the ecological importance of maintaining diverse forage assemblages in savanna systems. This is particularly true where fencing, land‐use change and habitat fragmentation constrain herbivore movements, increasing reliance on locally available forage (Osipova et al. 2018; Cassidy et al. 2013). Forage heterogeneity enables dietary flexibility and therefore supports mixed feeding strategies (Hofmann 1989; Kos et al. 2012; Kartzinel and Pringle 2020; Fynn and Provenza 2023; Botha et al. 2024).

African elephants ( *Loxodonta africana* ) and impalas ( *Aepyceros melampus* ) exemplify this mixed‐feeding strategy (Kos et al. 2012). Among the many ungulates of southern African savannas, this pair is especially informative. Both species are abundant and co‐occurring: impalas are the most numerous ungulate in the Greater Kruger system, while elephants dominate herbivore biomass (Abraham et al. 2019; Comley et al. 2025; Wessels et al. 2026). Additionally, they occupy opposite extremes of body size and digestive physiology among mixed feeders (Kos et al. 2012; Codron et al. 2006; Potter et al. 2022). This contrast makes them well suited for testing differences in overall diet composition under shared seasonal forage limitation. Their shared mixed‐feeding strategy is most evident in their seasonal dietary shifts. During the wet season, both species forage predominantly on grassy vegetation (Cerling et al. 2006; Meissner et al. 1996; Sponheimer et al. 2003; Kos et al. 2012; James et al. 2022). As the dry season progresses, reductions in grass quality increasingly favour a dietary shift toward browse (Van Soest 1994; Kos et al. 2012; Gill et al. 2023). However, browse is not without costs as it commonly contains secondary compounds like tannins that can limit intake and digestive efficiency, particularly in smaller ruminants such as impalas (Jansen et al. 2007; Kos et al. 2012). Despite sharing a mixed‐feeding strategy, elephants and impalas may adopt divergent foraging strategies to accommodate their contrasting physiological constraints (Kos et al. 2012; Potter et al. 2022).

Elephants tolerate high‐fibre diets through a strategy of quantity over quality. As large‐bodied hindgut fermenters, they sustain high intake rates and rapid gut transit to process vast quantities of coarse vegetation (Owen‐Smith 1988; Clauss et al. 2003). Impalas, in comparison, are small‐bodied ruminants with longer gut retention times and comparatively higher nutrient requirements. These characteristics reduce their ability to maintain ample dietary intake when relying on fibre‐ and tannin‐rich browse (Kos et al. 2012). Although impalas can partially offset these limitations through physiological plasticity, for example, through seasonal modulation of rumen papillae surface area, such compensatory responses are constrained and insufficient to fully defend them against declines in forage quality (Kos et al. 2012). Beyond digestive physiology, body size also shapes the spatial scale at which each species forages. Elephants range widely, consuming forage across broad landscape mosaics, whereas impalas are comparatively sedentary, foraging within small, stable home ranges (Wittemyer et al. 2007; Abraham et al. 2019). These contrasting scales of movement may expose each species to different subsets of the available vegetation. Overall, these differences in digestive physiology, body size and movement ecology are therefore expected to structure species‐specific dietary responses to seasonal resource limitation, with important consequences for resource partitioning and the intensity of interspecific competition (Kos et al. 2012; Kartzinel et al. 2015; Codron et al. 2006; Potter et al. 2022; Wang et al. 2025). This may drive divergent reliance on forbs versus woody browse between species, with impalas potentially favouring the former to mitigate physiological limitations. Previous studies suggest that some forb taxa retain relatively favourable nutritional characteristics during the late dry season due to high nutrient concentrations and potentially lower structural or chemical defences compared to most woody browse taxa (Dunham 1980; Kos et al. 2012; Sponheimer et al. 2003; Fritz et al. 2002; Fynn and Provenza 2023).

Understanding dry‐season diets of mixed feeders is crucial not only for evaluating their nutritional constraints but also for assessing their broader influence on dry‐season resource competition. Dietary flexibility can allow mixed feeders to persist at higher population densities than other feeding guilds, but that will potentially increase the pressure on shared fallback plant resources as forage quality declines (Staver and Hempson 2020). Furthermore, climate projections indicate an increase in both the frequency and severity of drought events (Ayugi et al. 2022; Chivangulula et al. 2025), which is likely to exacerbate seasonal forage limitation across savanna ecosystems (Abraham et al. 2019; Umuhoza et al. 2025). These dynamics are also increasingly affected by anthropogenic pressures. For instance, across many savanna systems, livestock grazing has altered vegetation structure and composition, commonly reducing grass cover, suppressing grazing‐intolerant forbs and modifying woody plant dynamics (Siebert and Dreber 2019; Dreber et al. 2011; Tessema et al. 2016). Fire is another intrinsic driver of savanna vegetation dynamics (Bond and Keeley 2005). Changes in the timing or frequency of fire, whether associated with land management, other human activities or increasing drought, may alter vegetation composition and forage heterogeneity (Archibald et al. 2012). Frequent burning may favour fire‐tolerant species, whereas fire suppression can promote woody encroachment (Case and Staver 2017; Wilcox et al. 2020). Fire can therefore modify the availability of standing forage and create patches of nutritious post‐fire regrowth, which may influence herbivore forage selection and dietary partitioning. All such vegetation changes may alter patterns of dietary partitioning and potentially intensify competitive interactions among herbivores. These effects may be especially pronounced among mixed feeders during the dry season when they become increasingly reliant on nutritionally important forage resources, such as forbs, because of constraints imposed by digestive physiology and body size. According to the Jarman–Bell principle, large‐bodied herbivores can tolerate more fibrous forage of lower quality and are therefore expected to have broader, less selective diets, whereas smaller‐bodied herbivores must select more nutrient‐rich and digestible forage to meet their relatively greater metabolic demands (Bell 1971; Jarman 1974). Identifying plant taxa consumed by elephants and impalas during the dry season can reveal which components continue to contribute to their diets when much of the herbaceous layer is senescent or dormant and hence be overlooked in conventional assessments of forage availability (Siebert et al. 2024). Loss of these nutritionally important forage resources could trigger cascading effects across other feeding guilds and compromise overall savanna resilience (Abraham et al. 2019).

Although numerous studies have documented broad seasonal dietary shifts in elephants and impalas, as well as large‐scale patterns of niche partitioning among sympatric savanna herbivores (Kartzinel et al. 2015; Codron et al. 2007; Meissner et al. 1996; Kos et al. 2012; Abraham et al. 2019, 2026), the specific plant taxa consumed, including potentially overlooked dry‐season forage resources such as forbs, remain poorly resolved. Much of the existing literature relies on traditional approaches such as microhistological or stable isotope analysis, which effectively characterise general dietary patterns but lack the taxonomic resolution needed to detect selectively consumed, low‐abundance or cryptic plant species (Pompanon et al. 2012; Valentini et al. 2009; Taberlet et al. 2012). Although some recent studies have applied metabarcoding to these specific herbivores (Kartzinel et al. 2015; Kartzinel and Pringle 2020; Gill et al. 2023; Abraham et al. 2026), most have used single‐locus markers, which offer improved resolution over traditional methods but remain vulnerable to marker‐specific bias (Deagle et al. 2019; Alberdi et al. 2018; Vallin et al. 2026). By contrast, dual‐locus metabarcoding has been shown to enhance taxonomic recovery and reduce bias, enabling more robust identification of plant taxa in complex diets (Botha et al. 2024; Alberdi et al. 2018; De Barba et al. 2014; Vallin et al. 2026). As a result, dry season dietary taxa may be systematically underrepresented in current dietary reconstructions.

In this study, we used dual‐locus DNA metabarcoding to quantify dry‐season diets of African elephants and impalas within the Timbavati Private Nature Reserve, part of the Greater Kruger National Park. To maximise taxonomic resolution and minimise regional assignment error, especially for locally diverse but globally underrepresented taxa, we utilised and expanded region‐specific plant reference databases encompassing locally occurring taxa (Botha et al. 2023, 2024; Pansu et al. 2019). We investigated whether the dietary composition of elephants' and impalas' differs in the late dry season, with particular emphasis on the plant taxa and functional groups (in this case, plant growth forms) underlying dietary differentiation between these co‐occurring mixed feeders. We tested the hypotheses that (i) elephants and impalas differ in dietary diversity and composition during the dry season and (ii) these differences are reflected in both the occurrence and relative contribution of plant taxa to their diets. By resolving species‐level dietary patterns within a shared landscape, this study identifies plant taxa that contribute disproportionally to the dry‐season diets of elephants and impalas and evaluates the contribution of woody, graminoid and forb taxa to their dietary differentiation. Identifying such candidate forage taxa can inform vegetation monitoring by drawing attention to resources that may be overlooked when habitat condition is assessed primarily through grass and tree dynamics. This broader perspective may support more functionally inclusive conservation, management and restoration of savanna vegetation under increasingly variable environmental conditions.

## 2 Materials and Methods

### 2.1 Study Site and Sample Collection

The study area used to characterise the dietary profiles of African elephants and impala is situated within the eastern semi‐arid savanna of Limpopo Province, South Africa, in the Lowveld Bioregion (Mucina and Rutherford 2006). Fieldwork was conducted in the Timbavati Private Nature Reserve (24°25′S, 31°20′ E), which covers approximately 53,400 ha and forms part of the Greater Kruger National Park conservation landscape, allowing elephants, impalas and other wildlife to move freely across the reserve boundaries into the Kruger National Park protected area. The region experiences a semi‐arid climate with a mean annual rainfall of 350–500 mm, most of which occurs during the summer months (October–March) (Shackleton 2000; Matsika et al. 2013; Phefadu and Kutu 2016; Munjonji et al. 2020). Fire occurrence varies spatially and temporally across the broader landscape. Vegetation in the study area is characterised as broad‐leaved savanna dominated by woody species such as *Senegalia nigrescens*, *Sclerocarya birrea* subsp. *caffra*, *Albizia harveyi* and members of the Combretaceae family (Mucina and Rutherford 2006). The herbaceous layer is primarily composed of perennial grasses, including *Digitaria eriantha* subsp. *eriantha*, *Eragrostis rigidior*, *Melinis repens* , *Schmidtia pappophoroides*, *Urochloa mosambicensis* and *Aristida congesta*, together with a diverse assemblage of forbs from genera such as *Commelina*, *Hibiscus* and *Sida*, and representatives of the Malvaceae, Fabaceae, Convolvulaceae and Acanthaceae families (Mucina and Rutherford 2006).

The vegetation description and floristic checklist used to construct the regional DNA reference libraries document the occurrence of plant taxa within the reserve. Vegetation surveys were not conducted concurrently with faecal sampling to provide estimates of their abundances in the landscape because reliable species‐level identification was constrained during the late dry season when most savanna plants lack diagnostic features, such as flowers. Faecal material from 24 individual elephants and 24 individual impalas was collected during the late dry season (October 2018), immediately prior to the onset of the first rains when forage availability is typically limited (Linstädter et al. 2013). Approximately 10 g of fresh faecal material was collected per individual immediately after defecation, homogenised and stored under sterile, cooled conditions for downstream molecular analyses. Sex and age class were not consistently recorded for both herbivore species and could therefore not be included as explanatory variables in the dietary analyses. Samples were collected across multiple herds to capture within‐species dietary variation in the reserve. Individual identity was confirmed through direct observation of defecation events or by ensuring adequate spatial and temporal separation between collections to avoid resampling the same individual. Collections were distributed across accessible areas of the reserve within central portions of extensive, relatively homogeneous vegetation units, rather than at a single locality or near boundaries with contrasting vegetation types. Exposure to recently burnt vegetation could not be assigned to each individual dietary sample or included as an explanatory variable. Sampling across multiple herds increased the likelihood of representing inter‐individual dietary variation, although herd identity and individual movement histories were not included as explanatory variables.

### 2.2 DNA Isolation, Amplification and Sequencing

DNA isolation and amplicon sequencing were done according to Botha et al. (2024). Approximately 200 mg of homogenised material from each faecal sample was used for DNA extraction with the Qiagen DNeasy Plant Mini Kit, following the manufacturer's instructions. Extraction blanks (PCR‐grade water) were included to monitor potential contamination. Chloroplastic *rbc*L and *trn*L (UAA intron) regions were amplified using the primer pairs *rbc*La‐f/*rbc*La‐r (Levin et al. 2003; Kress and Erickson 2007) and *trn*Lc/*trn*Ld (Taberlet et al. 1991) (primer sequences available in Appendix 1, Table A1). For library preparation, samples were dual‐indexed using the Illumina Nextera XT Index Kit, allowing all samples to be pooled for sequencing (multiplexing). Equimolar pools of indexed libraries were denatured and diluted according to Illumina specifications, spiked with PhiX control and sequenced on the Illumina MiSeq platform (2 × 300 bp), generating FASTQ files for downstream analysis. All amplicon sequencing was done by NICD Sequencing Core Facility (Pretoria, South Africa). For both the *rbc*L and *trn*L markers, impala samples were numbered 25–48 with a control sample labelled C1 and elephant samples 49–72, with control sample labelled C3, yielding a total of 200 raw FASTQ files.

### 2.3 Bioinformatics Processing and Taxonomic Assignment

Raw sequence data were processed using a validated metabarcoding workflow designed to maximise taxonomic resolution while minimising sequencing artefacts. The analytical framework followed the pipeline benchmarked by Botha (2023), which was validated using mock communities and applied to herbivore dietary datasets, demonstrating high precision in plant species identification. In the present study, the same core components (DADA2‐based denoising, abundance and frequency filtering and LULU post‐clustering curation) were implemented within a reproducible QIIME2 and Python‐based environment.

Initial quality control and trimming steps involved the removal of primer sequences using Cutadapt (v5.0; Martin 2011), allowing for a maximum mismatch rate of 10%, and the discarding of reads containing ambiguous nucleotides and < 50 bp after trimming. Sequence quality was assessed before and after trimming using FastQC and summarised across samples with MultiQC (Ewels et al. 2016). Following this assessment, all subsequent analyses were based on unmerged forward reads, as forward and reverse reads could not be merged due to insufficient overlap (≥ 20 bp required by DADA2). This is a common limitation for long or variable amplicons such as *rbc*L and the *trn*L intron (Pauvert et al. 2019; Tan et al. 2019). In order to infer amplicon sequence variants (ASVs), the DADA2 denoise‐single algorithm was implemented in QIIME2 (qiime2‐amplicon‐2024.10, Bolyen et al. 2019; Callahan et al. 2016), applying a maximum expected error threshold of 2, truncation at the first position with a Phred score < 20, and pooled chimaera detection. ASVs shorter than 50 bp were then removed. Resulting ASVs were further refined using the LULU post‐clustering algorithm (Frøslev et al. 2017) to merge putative erroneous daughter sequences based on similarity and co‐occurrence patterns. Frequency‐based filtering was then applied to both the *rbc*L and *trn*L datasets, removing ASVs detected in only one sample. In addition, an abundance threshold of 0.005% of total reads was applied exclusively to the *rbc*L dataset.

Taxonomic assignment was performed using region‐specific reference libraries for the *rbc*L and *trn*L (UAA intron) markers derived from the validated DNA barcode databases developed by Botha et al. (2023) for the study area. These reference libraries were originally constructed from a comprehensive floristic checklist of plant species occurring within the reserve. The *rbc*L library comprised 1238 sequences representing 318 genera and 562 species, while the *trn*L library contained 921 sequences representing 270 genera and 461 species.

To maximise representation of locally occurring taxa, the floristic checklist used to construct the original databases was systematically cross‐referenced against the corresponding *rbc*L and *trn*L reference libraries to identify species for which barcode sequences were unavailable at the time of database compilation. Newly available accessions for these missing taxa were subsequently retrieved from GenBank and incorporated into the respective marker libraries, following the quality‐control criteria outlined in Botha (2023). In total, 58 accessions were added (44 *rbc*L, 14 *trn*L), newly representing 27 and 14 species in the respective libraries. Relative to the floristic species list for the study area (Botha et al. 2023), the expanded *rbc*L and *trn*L libraries represented 89.4% and 79.9% of listed species at genus level or better, respectively, and in combination covered 87.2% of species at species level and 96.2% at genus level or better. The curated ASVs were then queried against the expanded libraries using BLASTn (v2.12.0; Camacho et al. 2009), applying a minimum identity threshold of 80% and e‐value ≤ 1e−10, and retaining up to three top hits per query. Taxonomic assignments were resolved to genus or species level where possible through expert evaluation of BLAST statistics (percentage identity, query coverage and e‐values) in conjunction with ecological knowledge of the study area.

Final taxonomic assignments were merged with DADA2 feature tables to generate diet matrices for each consumer species and barcode marker. Control samples were screened for potential contamination, and ASVs that occurred exclusively in controls or had higher relative abundance in controls than in biological samples were removed. As a final quality‐control step, any remaining unassigned ASVs were queried against the NCBI nt database, and non‐plant matches were excluded to ensure that only ecologically plausible dietary taxa were retained.

To assess taxonomic assignment performance and quantify the proportion of unassigned (NA) reads across samples, relative read abundance (RRA) and frequency of occurrence (FOO) metrics were calculated following assignment. RRA was expressed as the percentage of reads assigned to each taxon relative to the total assigned reads per sample, while FOO represented the percentage of samples in which each taxon was detected. These metrics enabled evaluation of the distribution and prevalence of NA reads, thereby providing an assessment of overall database resolution prior to downstream ecological analyses. Detailed per‐sample RRA and FOO summary plots, including NA reads, are provided in Figures A1, A2, A3, A4, A5, A6.

### 2.4 Statistical Analyses

All statistical analyses and visualisations were conducted in Python. However, for specific tasks that lacked native Python packages and support (e.g., DESeq2), R was implemented. To compare the dietary profiles of impalas and elephants, four distinct diet lists, categorised by herbivore and molecular marker (*rbc*L and *trn*L) were utilised. Each of these datasets contained taxonomically assigned ASVs with total read abundances per sample. Furthermore, each marker was analysed separately to generate complementary dietary profiles for each animal, as combining results across markers is not feasible due to marker‐specific amplification biases. While combining datasets may have been possible for presence‐absence data, it is often not appropriate for analyses using normalised proportional data (Da Silva et al. 2019).

Firstly, to evaluate dietary diversity, alpha diversity was quantified using the Shannon index (Shannon 1948) calculated from RRA matrices of taxonomically assigned ASVs. For each dataset (elephant and impala, *rbc*L and *trn*L), Shannon indices were computed at the sample level using the scikit‐bio library in Python (Aton et al. 2026). The resulting indices were aggregated by consumer species and barcode for comparative analyses. Furthermore, normality of Shannon indices was assessed using the Shapiro–Wilk test (SciPy library; Virtanen et al. 2020). The data did not meet the assumptions of normality, and the Mann–Whitney U test (for non‐parametric data) was applied to compare significant differences in dietary diversity between elephants and impalas, with significance set at *p* < 0.05. Data visualisation was then performed using Seaborn and Matplotlib (Waskom 2021; Hunter 2007) to generate boxplots with overlaid strip plots, annotated with corresponding *p*‐values to illustrate statistical differences.

To assess overall dietary structure and similarity between elephants and impalas, we evaluated beta‐diversity using two complementary approaches. First, presence‐absence (FOO) matrices were used to calculate Jaccard distances to capture compositional differences independent of abundance; these were visualised via non‐metric multidimensional scaling (NMDS) ordinations (scikit‐learn and scikit‐bio packages; Pedregosa et al. 2011; Aton et al. 2026; Jaccard 1912). Second, to incorporate the proportional importance of taxa, we calculated Bray‐Curtis dissimilarities and Pianka's niche overlap index from RRA matrices (Pianka 1973). The latter was implemented as cosine similarity via scikit‐learn, which provided a quantitative measure of resource overlap between individual diets. Statistical significance of dietary separation was tested using PERMANOVA and ANOSIM (scikit‐bio). Because PERMANOVA can be sensitive to differences in within‐group dispersion (Anderson 2001; Warton et al. 2012), homogeneity of multivariate dispersion was assessed using PERMDISP implemented in scikit‐bio for each marker. PERMDISP tested whether elephant and impala samples differed in their average distance to the group centroid, allowing PERMANOVA results to be interpreted as compositional differences only when dispersion was not significantly different between groups. Mann–Whitney U tests (SciPy) were then used to compare within‐ versus between‐species Pianka overlap values. All visualisations were generated using Matplotlib and Seaborn. While primary ordinations utilised Jaccard distances, corresponding RRA‐based Bray‐Curtis NMDS plots were provided in Appendix 1, Figure A7. We acknowledge that RRA is an imperfect proxy for the amount of each taxon consumed, as equal consumption of two plant taxa may not yield equal read abundance. Such discrepancies can arise from marker‐specific amplification biases and differential digestibility among plant taxa, which may differ further between species with contrasting digestive physiologies (Alberdi et al. 2018; Pompanon et al. 2012; Deagle et al. 2019; Botha et al. 2024; Vallin et al. 2026); however, we included these analyses to provide a more nuanced view of dietary dominance. The congruence between these RRA‐weighted results and our conservative FOO‐based patterns serves as crucial cross‐verification. This ensures that the identified dietary partitioning reflects a robust biological signal rather than an artefact of data transformation.

To visualise dietary composition, dominance patterns (FOO%) and dietary differentiation between herbivore species, FOO‐based Sankey diagrams were generated using the Plotly package (Kruchten et al. 2026). For each plant genus, FOO was calculated as the percentage of faecal samples in which that genus was detected per herbivore species, with only genera present in at least one sample retained (unassigned ASVs were excluded). Links between herbivore species and plant genera were weighted by FOO%, while nodes were colour‐coded by plant life form (graminoid, woody taxa, succulent, forb), enabling visual comparison of dietary composition and dominance patterns. Corresponding Sankey diagrams based on relative read abundance (RRA) are provided in Appendix 1, Figure A8.

To identify plant genera significantly associated with each herbivore, we implemented differential abundance testing via DESeq2 in R (Love et al. 2014). This approach identified plant genera consumed in significantly different proportions by one herbivore species relative to the other. Raw ASV abundances were collapsed to the genus level, and size factors were estimated using the poscounts method to account for the sparsity of metabarcoding data. Genera with |log_2_ fold change| > 1 and Benjamini‐Hochberg‐adjusted *p*‐values < 0.05 were considered significantly enriched. Differentially abundant genera were visualised with volcano plots using ggplot2 package in R (Wickham 2016).

## 3 Results

### 3.1 Metabarcoding Sequence Summary and Reference Database Evaluation

Illumina MiSeq sequencing yielded 10,137,912 *rbc*L and 10,723,495 *trn*L reads across all samples. Following DADA2 denoising, LULU curation, and stringent abundance filtering, the dataset was reduced to 244 *rbc*L and 697 *trn*L ASVs retained across all elephant and impala samples. During quality control, one impala individual (Sample 48) was excluded from all downstream analyses due to insufficient read depth across both markers. In addition, 260 non‐plant ASVs (primarily bacterial and viral sequences) were removed following NCBI *nt* database verification. A further four ASVs were excluded after being detected in negative controls or exhibiting relative abundance patterns indicative of low‐level laboratory contamination. The resulting dataset formed the final diet matrices used for analysis.

Taxonomic assignment success varied by marker. Unassigned (NA) reads were detected in all samples but contributed more substantially to total RRA in the *rbc*L datasets (elephant: 33.8%; impala: 19.9%) than in the *trn*L datasets (elephant: 8.5%; impala: 3.0%). Notably, while all impala samples yielded successful taxonomic assignments, incomplete assignments were observed in three elephant samples (51, 56, 57 for *rbc*L; and 56, 61, 62 for *trn*L), which resulted exclusively in unassigned reads after filtering. Detailed per‐sample ASV compositions, RRA and FOO values are provided in Figures A1, A2, A3, A4, A5, A6.

### 3.2 Dietary Diversity and Overlap Between Consumers

Dietary alpha diversity, quantified via the Shannon index, revealed no significant differences between elephants and impalas for either barcode marker (Figure 1). For the *rbc*L marker, elephants exhibited higher inter‐sample variability in diversity index values, yet median diversity remained comparable to that of impalas (*p* = 0.934). Similarly, *trn*L data indicated a marginal trend toward higher median diversity in impalas, though it was not statistically significant (*p* = 0.672). Collectively, these results demonstrate that both herbivores maintained comparable dietary breadths, with similar levels of richness and evenness in the plant taxa consumed across both markers.

**FIGURE 1:**
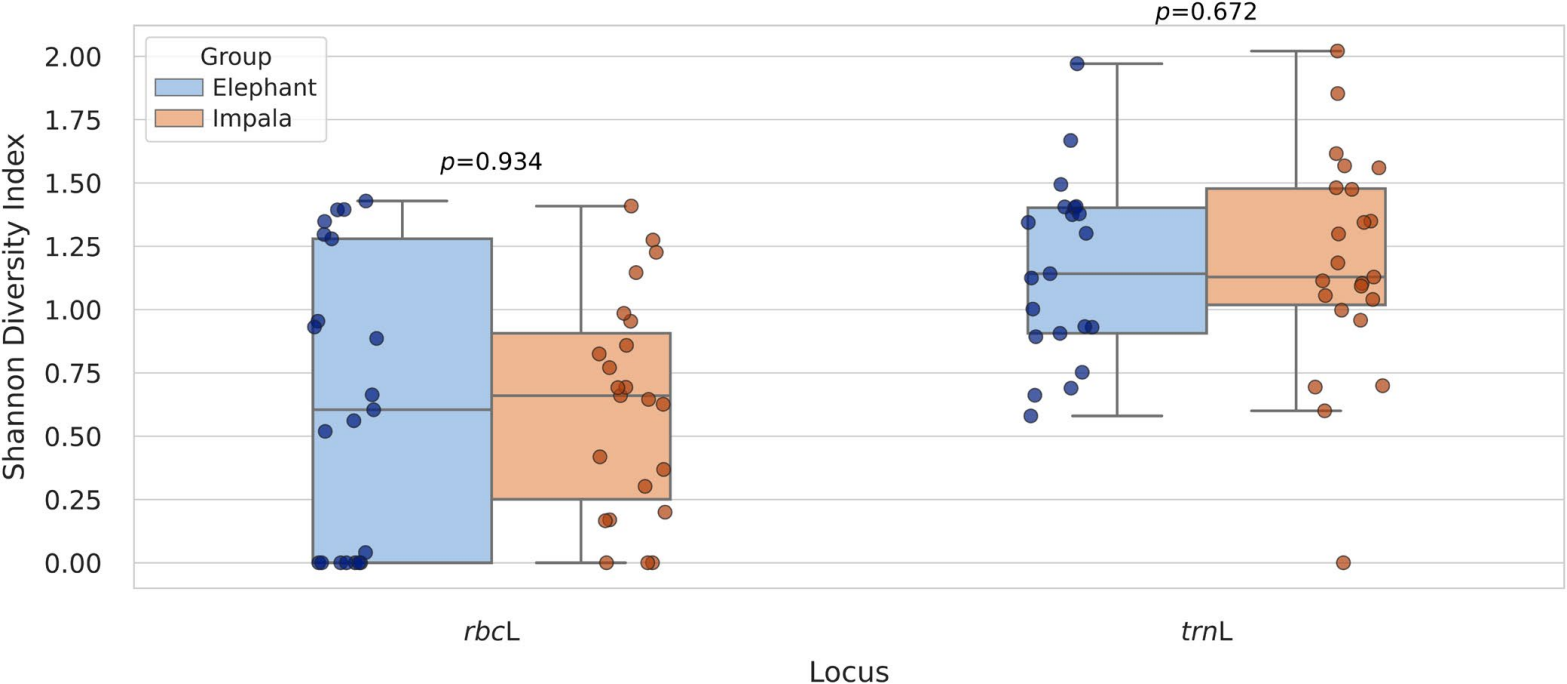
Comparison of dietary alpha diversity (Shannon index) between African elephants and impalas based on *rbc*L and *trn*L chloroplast DNA loci. Boxplots show Shannon diversity indices calculated from relative read abundance (RRA) data (*rbc*L and *trn*L: elephant *n* = 21, impala *n* = 23). Boxes represent interquartile ranges with medians indicated by horizontal lines, and individual samples shown as jittered points. *p*‐values from Mann–Whitney U tests are shown above each locus.

Beta‐diversity analysis using presence‐absence (FOO) based Jaccard distances revealed significant differences in dietary composition between elephants and impalas, with stronger separation observed for the *trn*L marker than for *rbc*L (Figure 2). For *rbc*L, PERMANOVA results (pseudo‐F_1,42_ = 5.62, *p* = 0.001) and ANOSIM (*R* = 0.32, *p* = 0.001) indicated moderate but significant dietary segregation, although some overlap between species was evident (stress = 0.205). However, PERMDISP indicated significant heterogeneity of dispersion for *rbc*L (F_1,42_ = 15.06, *p* = 0.001), therefore suggesting that this pattern may partly reflect unequal within‐group dispersion. In contrast, the *trn*L marker showed much stronger divergence (PERMANOVA pseudo‐F_1,42_ = 10.61, *p =* 0.001; ANOSIM *R* = 0.81, *p =* 0.001), with more distinct clustering between species (stress = 0.210). PERMDISP was not significant for *trn*L (F_1,42_ = 1.37, *p* = 0.202). This indicates that the observed separation was not primarily driven by heterogeneity of dispersion. Across both markers, elephant samples exhibited greater multivariate dispersion than impala samples, indicating higher inter‐individual variation in diet, although this dispersion effect was statistically supported only for *rbc*L. These patterns were consistent with Pianka's niche overlap index derived from RRA data. For *rbc*L, within‐species overlap was moderate (elephants = 0.17; impalas = 0.47), while between‐species overlap was marginally lower (0.16). For *trn*L, however, within‐species overlap was higher (0.68), whereas between‐species overlap was minimal (0.009). Mann–Whitney U tests confirmed that between‐species overlap was significantly lower than within‐species overlap for both markers (*p* < 0.001), supporting a pattern of strong species‐specific dietary differentiation. These findings were further corroborated by RRA‐based Bray‐Curtis NMDS ordinations provided in Figure A7.

**FIGURE 2:**
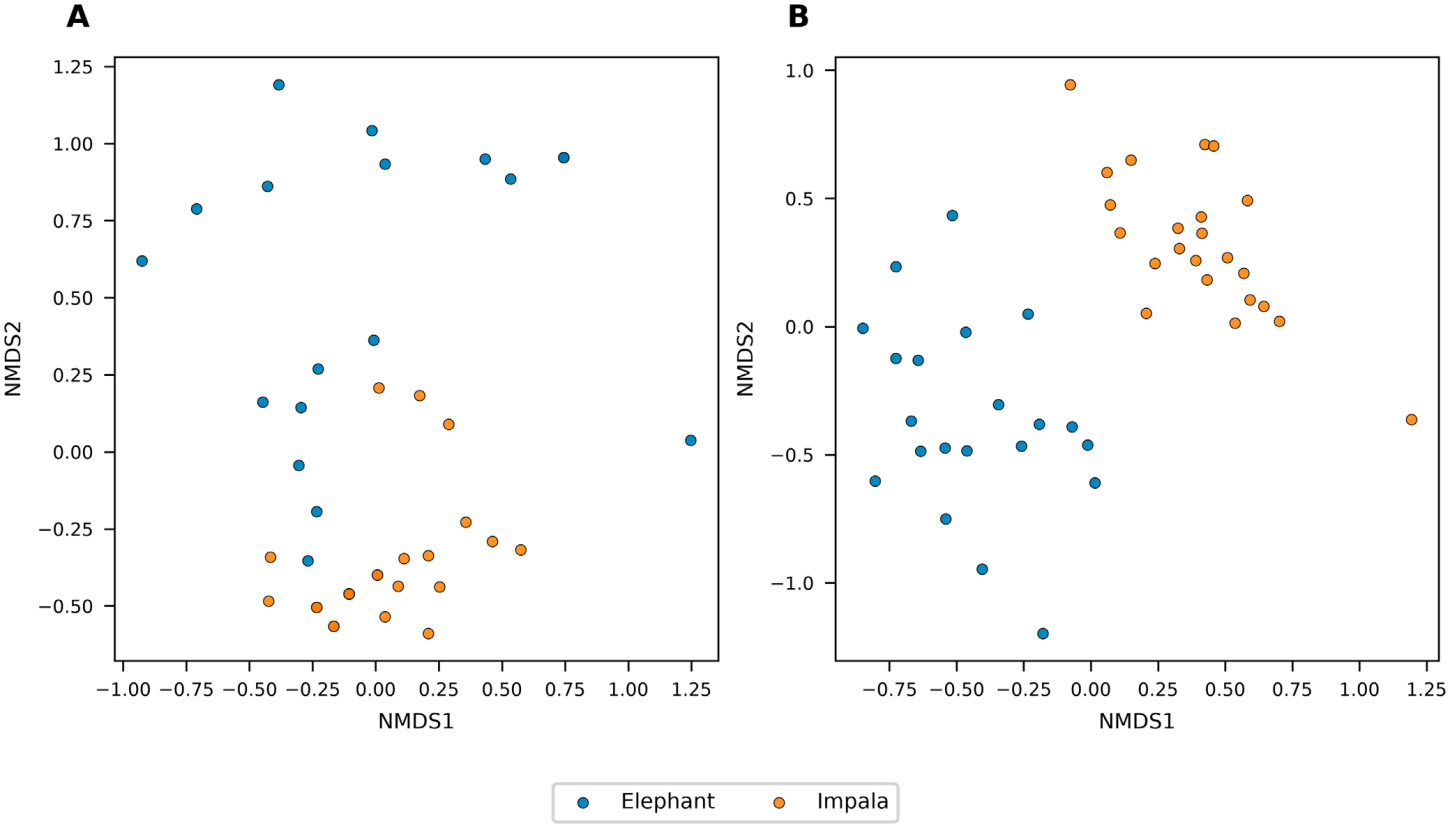
NMDS ordinations based on Jaccard distances of presence‐absence data showing dietary composition of elephants and impala for each DNA barcode marker. (A) *rbc*L and (B) *trn*L. Points represent individual faecal samples, coloured by animal (elephant: Blue; impala: Orange). (*rbc*L and *trn*L: Elephant *n* = 21, impala *n* = 23).

### 3.3 Dietary Composition and Differentiation Between Species

FOO‐based Sankey diagrams revealed pronounced dietary differentiation between elephants and impalas across both chloroplastic markers (Figure 3). Although a shared core of woody taxa nodes was present, most genera were asymmetrically distributed between consumers, indicating structured genus‐level dietary differentiation rather than broad dietary overlap. In the *rbc*L dataset, elephant diets were dominated by woody browse, particularly *Grewia* (47.6%), *Vachellia* (42.9%) and *Dichrostachys* (38.1%), all of which were absent from impala diets. Additional woody taxa, such as *Senna* (23.8%), were also restricted to elephant diets. In contrast, impala diets were characterised by strong graminoid representation, with genera such as *Megathyrsus* detected in 100% of samples, but also incorporated several woody and forb taxa not recovered in elephant diets, including *Carissa* (30.4%), *Schotia* (26.1%) and forbs such as *Abutilon* and *Asparagus* (13.0% each).

**FIGURE 3:**
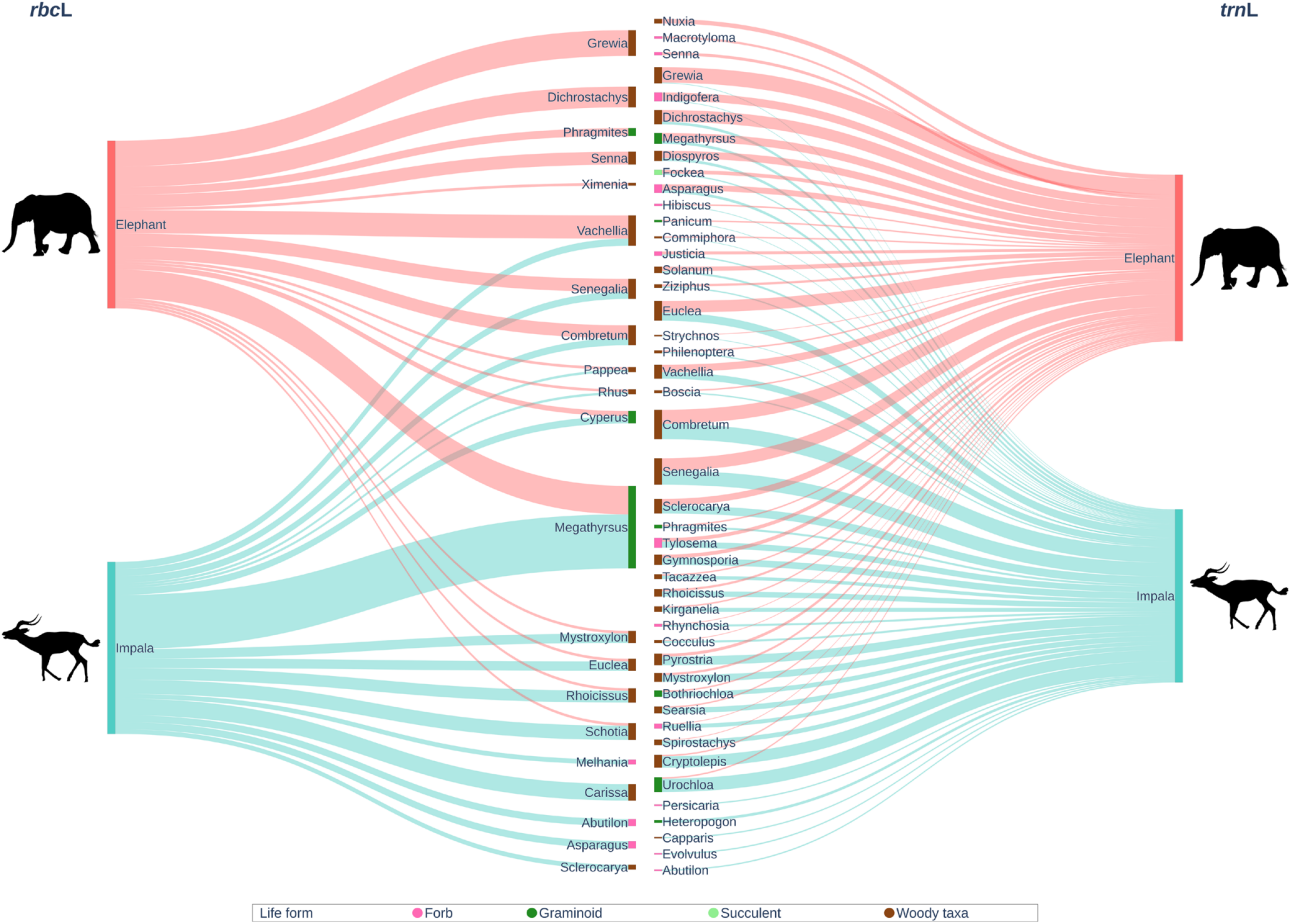
Frequency of occurrence (FOO%)‐based Sankey diagrams showing genus‐level dietary overlap between elephants and impalas for the *rbc*L (left) and *trn*L (right) datasets. Link width represents FOO%, indicating how often each genus occurred in the diet of each consumer. Plant genera are colour‐coded by life form (woody taxa, graminoid, forb or succulent). Wider links represent genera detected more frequently in the diets; shared connections indicate dietary overlap and exclusive links reflect genera detected in only one herbivore's diet (*rbc*L and *trn*L: elephant *n* = 21, impala *n* = 23).

The *trn*L marker revealed similar dietary patterns, but with higher taxonomic resolution, resulting in more pronounced differences. Elephant diets were overwhelmingly woody‐dominated, with near‐universal detection of *Grewia* (100%) and high frequencies of *Combretum* (90.5%), *Senegalia* (81.0%), *Dichrostachys* (76.2%) and *Euclea* (76.2%), alongside woody genera largely restricted to elephants such as *Nuxia* (28.6%) and forbs from the genus *Indigofera* (52.4%). Although impala diets also utilised shared woody taxa (e.g., *Combretum* 100%; *Senegalia* 91.3%), their diets showed markedly greater graminoid incorporation, with *Urochloa* occurring in 87.0% of impala samples but only 9.5% of elephant samples and *Bothriochloa* reaching 30.4%. Impala diets further exhibited higher frequencies of several woody genera (e.g., *Cryptolepis* 73.9%; *Pyrostria* 56.5%) and forbs such as *Tylosema* (39.1%) and *Ruellia* (26.1%). RRA‐based Sankey diagrams presented in Figure A8 corroborated these FOO patterns and confirmed asymmetric woody dominance in both consumers, but proportionally greater graminoid and forb contributions in impala diets. Detailed FOO percentages for all genera and species are provided in Appendix 1, Table A2.

Clear marker‐specific discrepancies were evident. For example, *Urochloa* was highly prevalent in impala diets in the *trn*L dataset (87.0%) but largely undetected by *rbc*L, whereas *Megathyrsus* was universally detected in impala diets using *rbc*L (100%) but occurred at a much lower frequency in the *trn*L dataset (17.4%). Similar inconsistencies were observed for certain other woody and forb taxa (e.g., *Asparagus*), thus demonstrating locus‐specific amplification bias and the value of the dual‐marker approach. Collectively, these results demonstrate consistent dietary differentiation structured along a woody‐graminoid‐forb axis, with elephants showing greater representation of woody browse taxa and impalas incorporating relatively higher proportions of graminoid and forb taxa during the dry season.

### 3.4 Differential Abundance of Dietary Genera

Differential abundance analysis using DESeq2 confirmed significant genus‐level divergence between elephant and impala diets (Figure 4; adjusted *p* < 0.05, |log_2_ fold change| > 1). In the *rbc*L dataset (Figure 4A), elephant diets were strongly enriched in woody browse taxa, with *Grewia* showing the largest effect size, followed by woody genera such as *Dichrostachys*, *Senna* and *Vachellia*, and the graminoid *Phragmites*. In contrast, impala diets were enriched in a broader mix of taxa, including woody genera (*Carissa*, *Rhoicissus*, *Euclea*, *Schotia*, *Mystroxylon*) and forbs (*Asparagus*, *Melhania*). The *trn*L dataset (Figure 4B) confirmed this pattern. Elephant diets again showed concentrated enrichment in woody browse, particularly *Grewia*, as well as other woody genera such as *Dichrostachys* and *Diospyros*, and the leguminous forb genus *Indigofera*. Impala diets, however, indicated significant enrichment across multiple functional groups, including woody taxa (*Spirostachys*, *Pyrostria*, *Cryptolepis*, *Combretum*) and the graminoid *Urochloa*. Overall, differential abundance analyses demonstrate that elephant diets show strong, high‐magnitude enrichment in a relatively narrow suite of woody browse taxa, whereas impala diets display enrichment across woody, graminoid and forb genera. These results quantitatively confirm dietary differentiation structured along functional group differences between the two herbivores.

**FIGURE 4:**
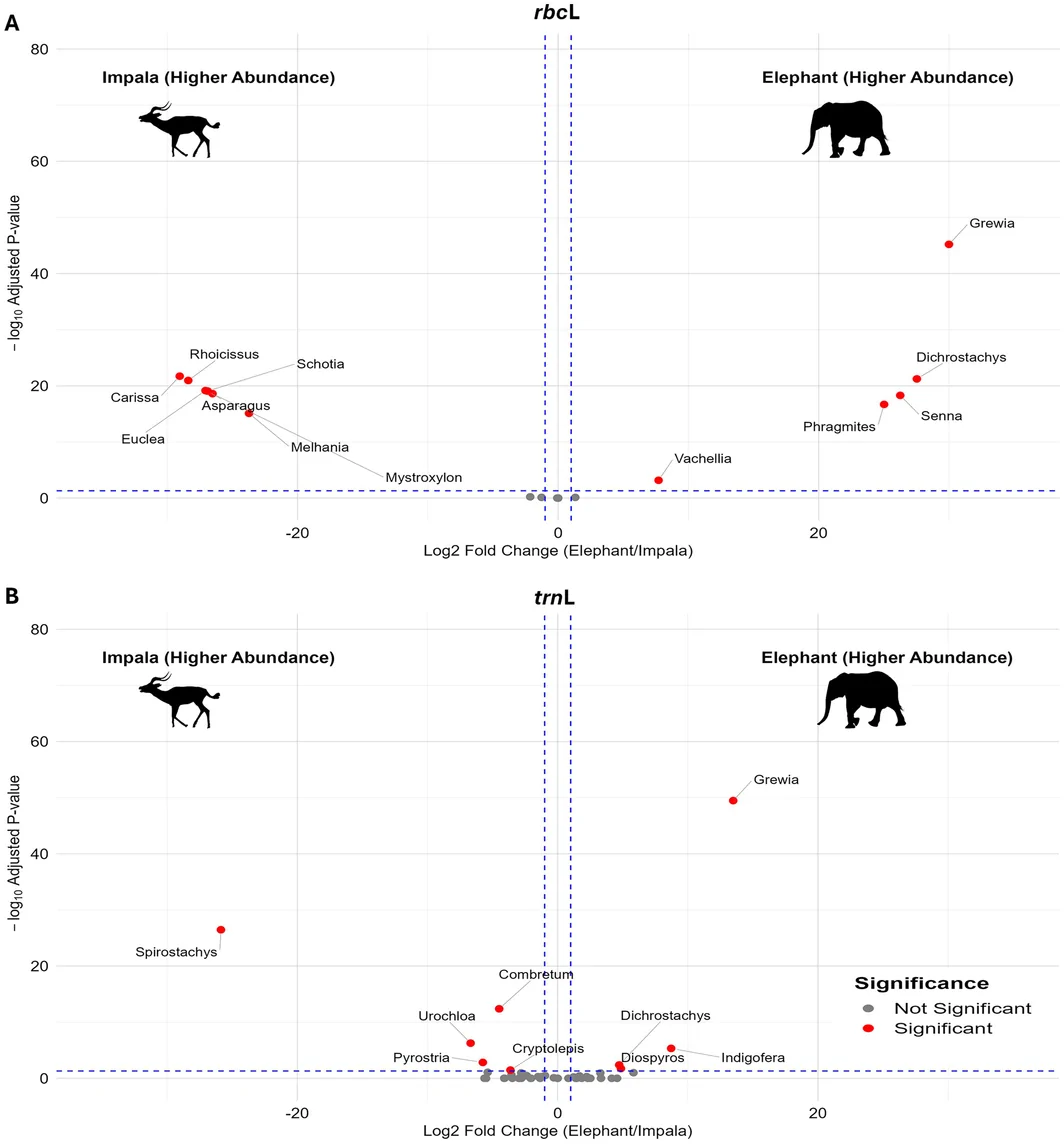
Volcano plots from DESeq2 analysis showing differential abundance of plant genera between elephant and impala diets for (A) *rbc*L and (B) *trn*L markers. The x‐axis represents log_2_ fold change (elephant/impala), with positive values indicating higher abundance in elephant diets. The y‐axis shows‐log_10_ adjusted *p*‐value. Red points denote significantly differentially abundant genera (adjusted *p* < 0.05, |log_2_ fold change| > 1); grey points were not significant. Labels highlight the most strongly enriched genera in each herbivore's diet.

## 4 Discussion

Our results demonstrate clear dry‐season dietary differentiation between elephants and impalas, despite both species functioning as mixed feeders within the same semi‐arid savanna landscape. A key finding of this study is that dietary alpha diversity (Shannon index) did not differ significantly between species (Figure 1), while beta‐diversity analyses consistently revealed strong compositional divergence and low dietary overlap, particularly in the *trn*L dataset. Similar patterns were observed for *rbc*L, although this marker was interpreted more cautiously because of marker‐specific dispersion effects. These findings suggest that dry‐season coexistence between the two herbivores is associated less with differences in overall dietary breadth and more with differences in the specific plant taxa incorporated into their diets.

The lack of divergence in dietary alpha diversity does not conform neatly to classical interpretations of the Jarman–Bell principle. However, increasing evidence suggests that body size alone is an inconsistent predictor of taxonomic dietary richness of free‐ranging herbivores (Kartzinel et al. 2015; Potter and Pringle 2023; Kartzinel and Pringle 2020; Potter et al. 2022; Wang et al. 2025). Instead, our findings support the view that herbivores with contrasting digestive physiologies may maintain comparable levels of dietary diversity while partitioning resources through differential use of plant taxa and functional groups. In this study, elephants and impalas consumed similarly diverse diets in terms of taxonomic richness, but the composition of those diets differed substantially.

The observed compositional divergence likely reflects the nutritional and structural constraints imposed by late dry season conditions, when high‐quality forage is limited. Under such conditions, mixed feeders may reduce dietary overlap through differential use of plant taxa and functional groups (Schoener 1974; Kos et al. 2012; Pansu et al. 2022). Compared with previous studies documenting dietary overlap among sympatric African herbivores (e.g., Kartzinel et al. 2015), the degree of dietary segregation observed here was relatively strong, particularly for the *trn*L marker. Although differences among ecosystems and sampling designs complicate direct comparisons, our findings are broadly consistent with evidence that dietary partitioning may intensify during periods of resource limitation and increased competition (Pansu et al. 2022). However, without corresponding vegetation‐availability data, we cannot determine the extent to which this divergence resulted from active selection or differences in the forage encountered by each herbivore.

Genus‐level Sankey plot (Figure 3) analysis further illustrated dietary differentiation. Elephant diets were consistently dominated by woody browse taxa, such as *Grewia*, *Vachellia* and *Dichrostachys*, whereas impala diets incorporated a broader mixture of woody taxa (*Carissa*, *Schotia*), forbs (*Asparagus*, *Melhania*) and graminoids (*Urochloa*). Differential abundance analyses reinforced this pattern by showing concentrated enrichment of a relatively narrow suite of woody browse taxa in elephants, while impala diets showed enrichment across multiple functional groups. Collectively, these findings suggest that elephants functioned primarily as woody browsers during the late dry season, whereas impalas maintained a more mixed feeding strategy with greater incorporation of graminoids and forb taxa. This differentiation is likely structured by a synergy of physiological and behavioural mechanisms (Kos et al. 2012; Hofmann 1989; Pansu et al. 2022; Potter et al. 2022).

These dietary differences are broadly consistent with expectations derived from digestive physiology and body size constraints (Belovsky 1978; Wang et al. 2025). As large‐bodied hindgut fermenters, elephants can maintain high intake rates and process large quantities of fibrous browse (Clauss et al. 2003; Owen‐Smith 1988). In contrast, smaller ruminants such as impala typically require more nutrient‐dense forage (Kos et al. 2012) and may be constrained by dense resins and essential oils characteristic of more heavily defended woody browse (Van Der Merwe and Marshal 2012; Siebert and Dreber 2019). For instance, woody taxa such as *Vachellia* and *Dichrostachys*, which were significantly enriched in elephant diets (Figure 4), possess high concentrations of tannins and oils that may be physiologically tolerable only to megaherbivores (Van Der Merwe and Marshal 2012). The relatively greater incorporation of graminoids and forb taxa in impala diets may therefore be consistent with dietary strategies that balance nutrient acquisition with digestive constraints. The contribution of forbs to impala diets is noteworthy. Several forb genera, including *Asparagus*, *Melhania*, *Tylosema* and *Ruellia*, were more frequently detected in impala diets relative to elephants. This suggests that forb taxa may contribute to dietary differentiation during the dry season, potentially by supplementing nutrient intake when grasses decline in quality. Nevertheless, woody browse remained the dominant component of dietary items for both herbivores, indicating that dietary differentiation was primarily structured by differential consumption of woody taxa, with graminoids and forbs contributing secondarily to this divergence. Taxon‐specific differences in the diet of elephant and impala were also reported in a semi‐arid Kenyan savanna, where forb taxa, specifically *Ruellia* spp., were selected by impalas and dik‐dik, but not by elephants, while *Grewia* spp. were selected by elephants and other browsers (Abraham et al. 2026). Differences in movement and feeding scale may also have contributed to the observed difference in dietary composition. Elephants range across broad landscape mosaics and can access spatially dispersed forage patches, whereas impalas generally forage within smaller home ranges and may encounter a more localised subset of the available vegetation (Wittemyer et al. 2007; Abraham et al. 2019). Consequently, differences in diet may reflect both species‐specific dietary requirements and differences in the plant assemblages encountered at their respective foraging scales. Spatial heterogeneity generated by vegetation structure, water distribution and recent fire may further influence these encounter rates. Because concurrent forage availability and the recent fire histories of the areas used by sampled individuals were not quantified, these factors should be regarded as alternative, non‐exclusive explanations for the observed dietary differences.

The application of a dual‐locus metabarcoding framework was instrumental in reducing the limitations associated with primer‐related biases and improving dietary resolution (Alberdi et al. 2018; Vallin et al. 2026; Botha et al. 2024). Our results demonstrate that *rbc*L and *trn*L provided complementary coverage, resolving taxa, such as *Megathyrsus* and *Urochloa* that may have been overlooked in a single‐marker study. This complementarity was also evident in marker‐dependent assignment success, where several elephant samples yielded exclusively unassigned reads for one marker but were successfully resolved by the other, highlighting the risk of taxon dropout in single‐locus studies (Corse et al. 2019; Shirazi et al. 2021). Nevertheless, the two markers did not produce identical signals. For *rbc*L, the significant PERMDISP result indicates that the PERMANOVA result may partly reflect unequal within‐group variability, rather than differences in dietary composition alone (Anderson 2001; Warton et al. 2012). This unequal distribution was driven primarily by greater variability among elephant samples. Biologically, this pattern could reflect inter‐individual differences or variation in the vegetation encountered by elephants moving through different parts of the landscape. Unmeasured differences in sex and age may also have contributed to this variation through their effects on body size, nutritional requirements, movement behaviour and physical access to forage. Alternatively, the observed variability may reflect marker‐specific coverage, amplification efficiency or primer bias (Corse et al. 2019; Shirazi et al. 2021; Alberdi et al. 2018). The greater dispersion in the *rbc*L dataset should therefore not be attributed solely to biological variation. Nevertheless, dietary differentiation between elephants and impalas was supported by the *trn*L beta‐diversity result, low Pianka overlap, consistent FOO‐ and RRA‐based dietary patterns, and DESeq2 genus‐level differences between consumers. Collectively, these complementary lines of evidence indicate that the observed dietary differentiation is unlikely to be solely a bioinformatic artefact.

Despite the high resolution provided, several limitations warrant consideration. First, the study represents a single late dry‐season snapshot and therefore cannot resolve how dietary overlap changes across seasons or under varying rainfall conditions. Second, plant availability surveys could not be conducted since the vegetation condition during the late dry‐season sampling constrained reliable species‐level identifications. As such, the ability to quantify the availability and abundance of foraged items was limited. The observed patterns therefore demonstrate dietary differentiation between elephants and impalas without clear evidence of selection differences between the two herbivore species. Furthermore, metabarcoding data provide information on dietary composition but not direct estimates of intake or consumed biomass. Consequently, conclusions regarding selective feeding strategies to balance nutrient acquisition remain inferential. Finally, although the use of two genetic loci substantially improved taxonomic resolution, some taxa remained unresolved. Future studies integrating metabarcoding with vegetation availability surveys, nutritional analyses, seasonal sampling and behavioural observations would provide a more mechanistic understanding of how savanna herbivores differentiate their use of resources under fluctuating environmental conditions (e.g., Abraham et al. 2026).

The taxonomic resolution achieved here also has practical relevance for savanna management. Broad assessments of grass condition and woody cover may overlook individual plant taxa that contribute substantially to herbivore diets, particularly forbs and less conspicuous woody species. The taxa identified in this study therefore represent candidate dry‐season forage resources that could be incorporated into vegetation monitoring and evaluated further in relation to their availability, phenology and nutritional value. The value of integrating these sources of information was demonstrated in a semi‐arid savanna in Kenya, where taxa that were strongly selected by elephants increased in abundance following the exclusion of these megaherbivores (Abraham et al. 2026). Tracking species‐specific forage resources could help determine whether management practices such as prescribed burning, herbivore‐density regulation or vegetation restoration maintain a sufficiently heterogeneous forage base for herbivores with contrasting dietary requirements. Nevertheless, the present findings should not be used to assign management priority to particular plant taxa until their dietary importance has been assessed relative to their availability in the landscape.

## 5 Conclusion

This study demonstrates pronounced dry‐season dietary differentiation between elephants and impalas within a shared savanna landscape. Although both herbivores exhibited comparable levels of dietary diversity, they differed strongly in dietary composition with elephants relying predominantly on woody browse taxa, while impalas incorporated a broader mixture of woody, graminoid, and in particular forb resources. This fine‐scale taxonomic differentiation could reduce dietary overlap between sympatric mixed feeders during periods of forage limitation. Our results further demonstrate the value of dual‐locus DNA metabarcoding for resolving complex herbivore diets and identifying taxonomic patterns that may be overlooked using single‐marker approaches. While woody browse formed the primary basis of dietary divergence, several graminoid and forb taxa also contributed to differentiation, particularly within impala diets. These findings suggest that taxonomic and functional forage heterogeneity may contribute to dietary flexibility and resource partitioning among savanna herbivores. Under increasing climatic variability and anthropogenic pressure, reductions or losses of particular dry‐season forage taxa could reduce opportunities for dietary differentiation and increase overlap in the resources used by co‐occurring herbivores. Savanna monitoring and management should therefore consider the diversity and composition of both woody and herbaceous vegetation, including forb taxa that may be overlooked in assessments centred primarily on trees and grasses.

## Author Contributions

**Kyle R. van Wyk:** data curation (lead), formal analysis (lead), investigation (equal), methodology (equal), software (lead), visualization (lead), writing – original draft (lead), writing – review and editing (equal). **Danielle Botha:** data curation (equal), methodology (equal), resources (equal), software (supporting), supervision (supporting), writing – review and editing (equal). **Sandra Barnard:** conceptualization (equal), investigation (equal), methodology (equal), resources (lead), supervision (lead), writing – review and editing (equal). **Frances Siebert:** conceptualization (equal), investigation (equal), methodology (supporting), resources (equal), supervision (supporting), validation (lead), writing – review and editing (equal).

## Funding

We thank the North‐West University for its support of this research.

## Ethics Statement

Animal research ethics approval for this study was granted by the North‐West University Animal Production Sciences Research Ethics Committee (NWU‐AnimProdREC) on 15 November 2018 (ethics number: NWU‐00177‐18‐A5). All research was conducted in strict accordance with the conditions stipulated under this approval.

## Conflicts of Interest

The authors declare no conflicts of interest.

## Figures and Tables

**FIGURE A1:**
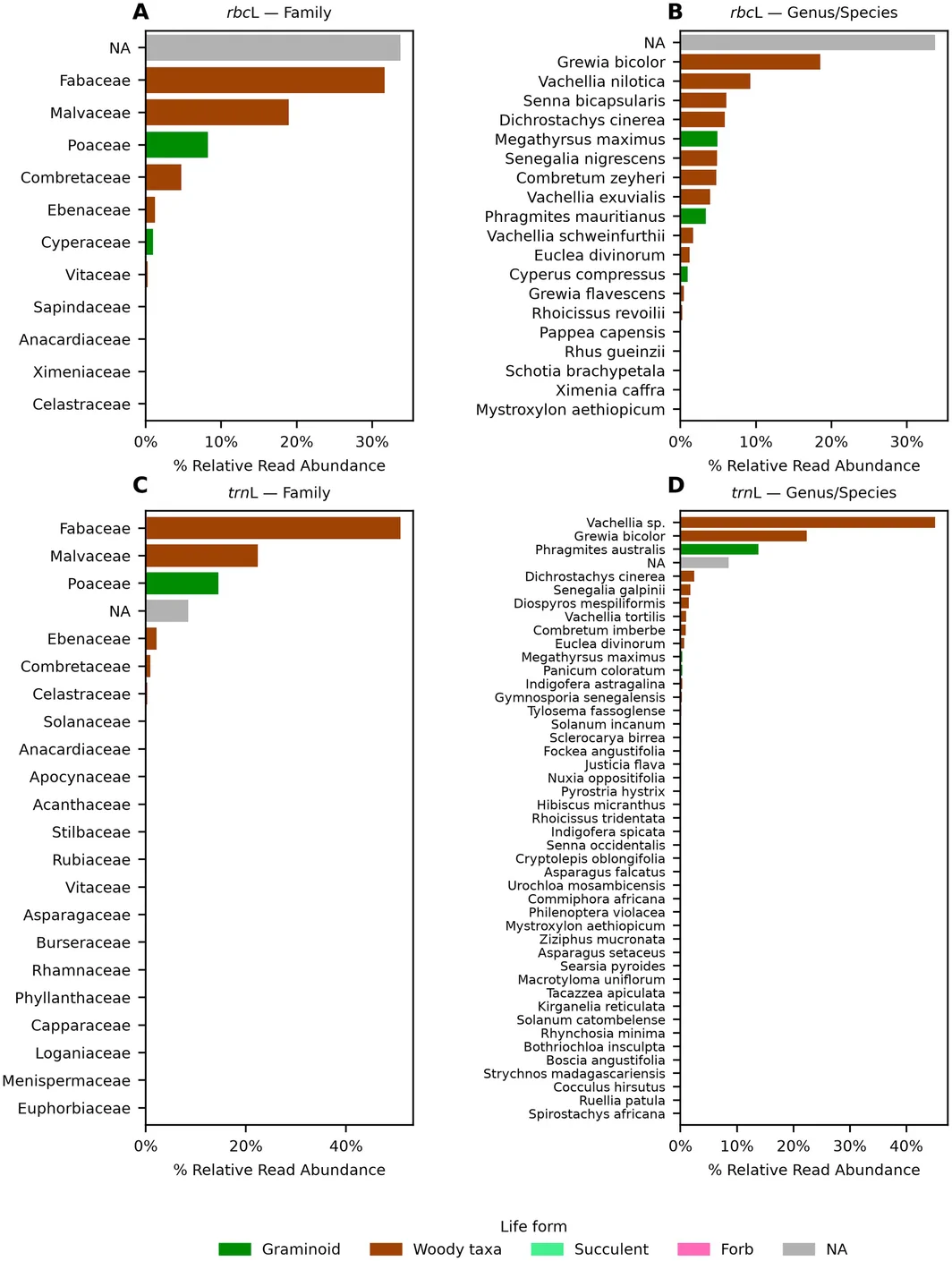
Relative read abundance percentage (RRA%) of plant taxa detected in elephant faecal samples using the *rbc*L and *trn*L markers. Panels A and B show the proportional composition of taxa detected with *rbc*L at the family and genus/species levels, respectively, while panels C and D show the corresponding *trn*L results. Bars represent the percentage contribution of each taxon to total reads and are coloured by plant life form: woody taxa, graminoids, forbs and succulents. NA indicates unassigned ASVs.

**FIGURE A2:**
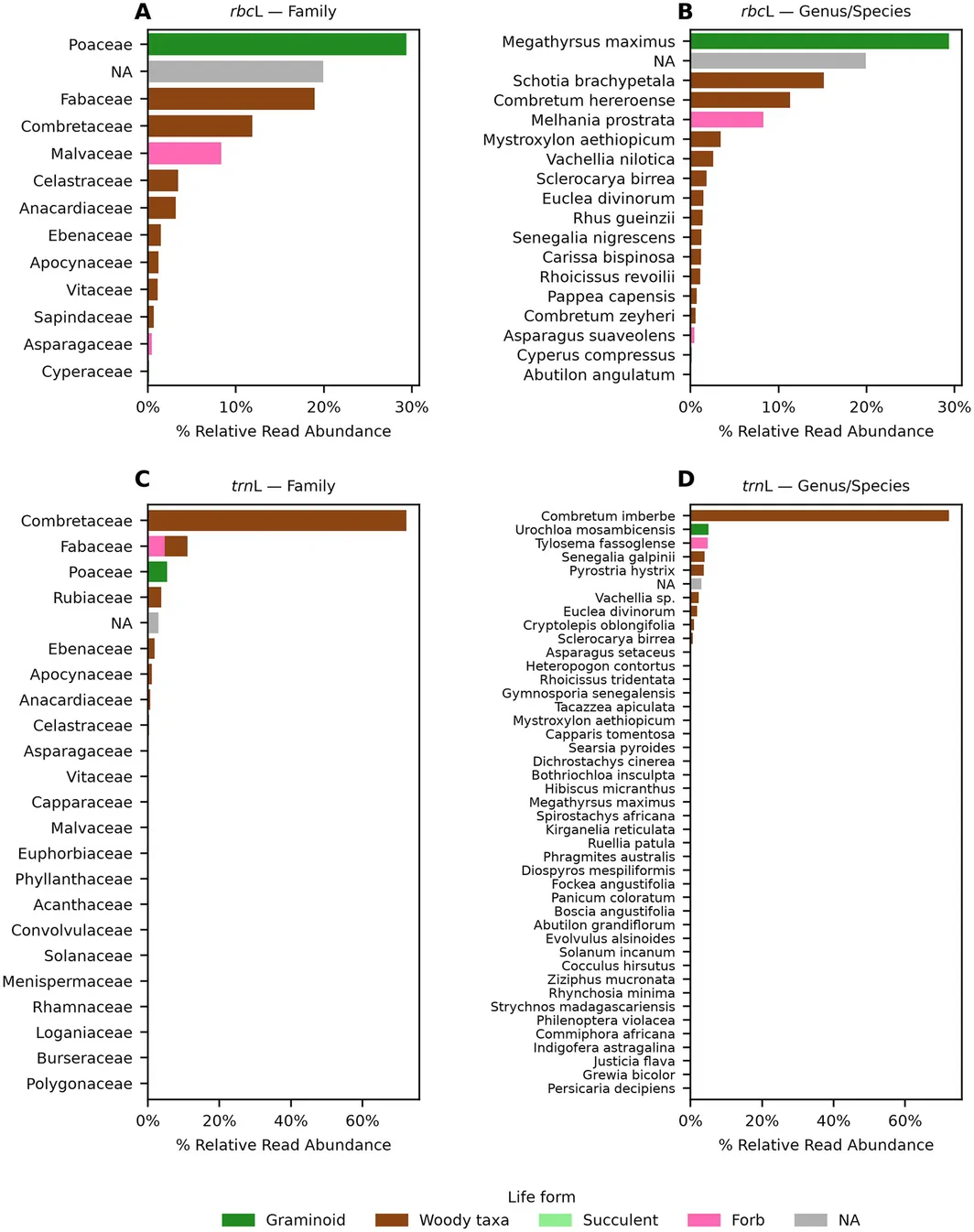
Relative read abundance percentage (RRA%) of plant taxa detected in impala faecal samples using the *rbc*L and *trn*L markers. Panels A and B show the proportional composition of taxa detected with *rbc*L at the family and genus/species levels, respectively, while panels C and D show the corresponding *trn*L results. Bars represent the percentage contribution of each taxon to total reads and are coloured by plant life form: woody taxa, graminoids, forbs and succulents. NA indicates unassigned ASVs.

**FIGURE A3:**
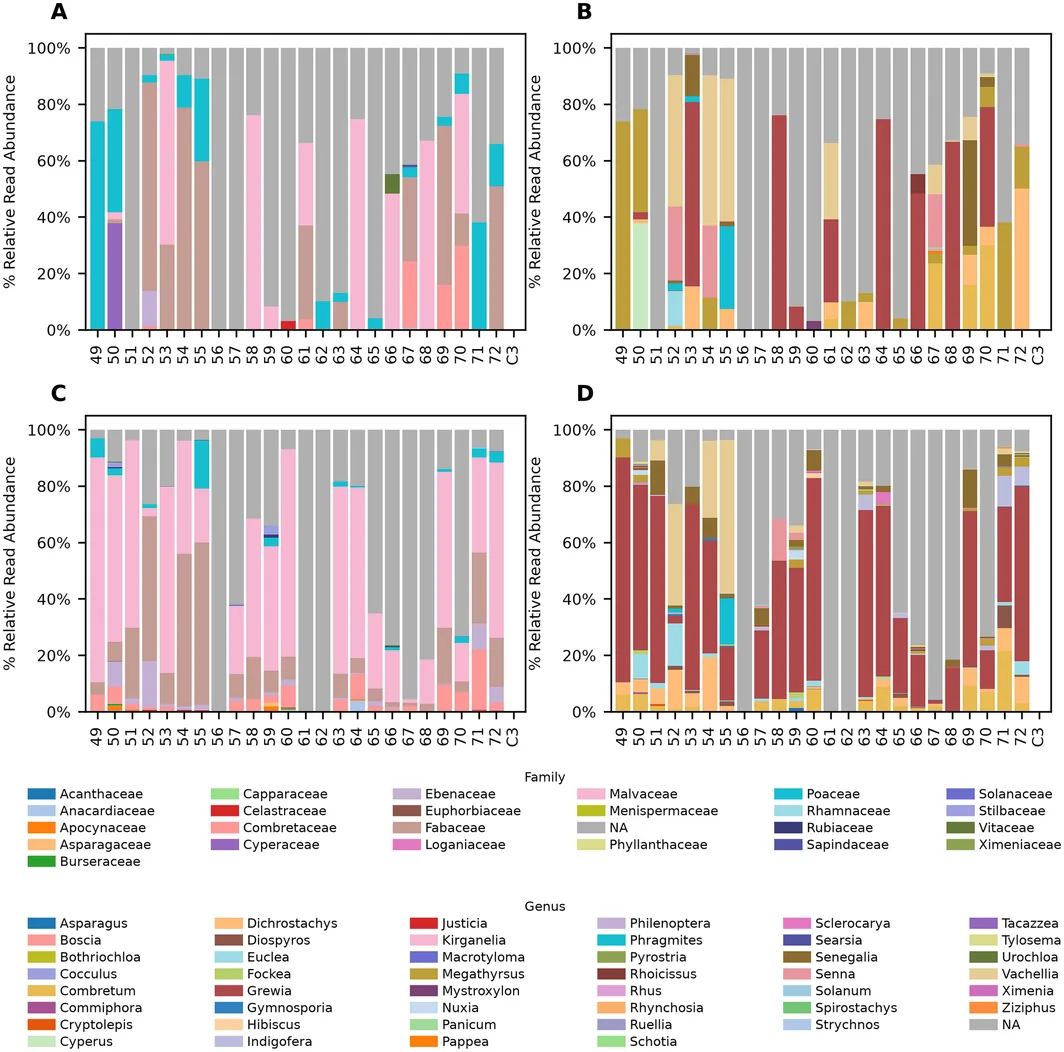
Relative read abundance (RRA) 100% stacked bar plots showing plant taxa detected in elephant faecal samples using the *rbc*L and *trn*L markers. Panels A and B show family‐ and genus‐level compositions for the *rbc*L dataset, respectively, while panels C and D show the corresponding *trn*L results. Bars indicate the proportional contribution of each identified taxon to total plant DNA reads per sample.

**FIGURE A4:**
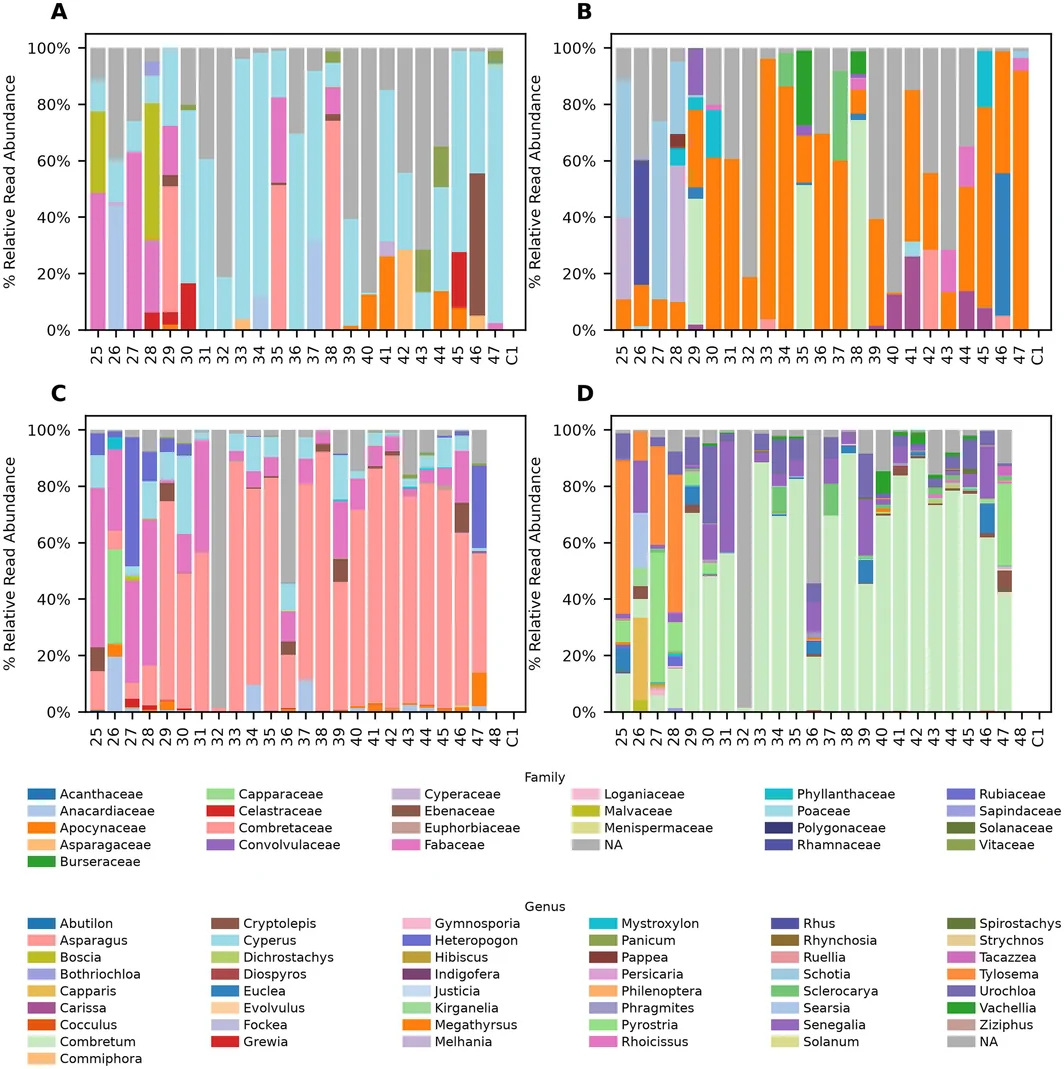
Relative read abundance (RRA) 100% stacked bar plots showing plant taxa detected in impala faecal samples using the *rbc*L and *trn*L markers. Panels A and B show family‐ and genus‐level compositions for the *rbc*L dataset, respectively, while panels C and D show the corresponding *trn*L results. Bars indicate the proportional contribution of each identified taxon to total plant DNA reads per sample.

**FIGURE A5:**
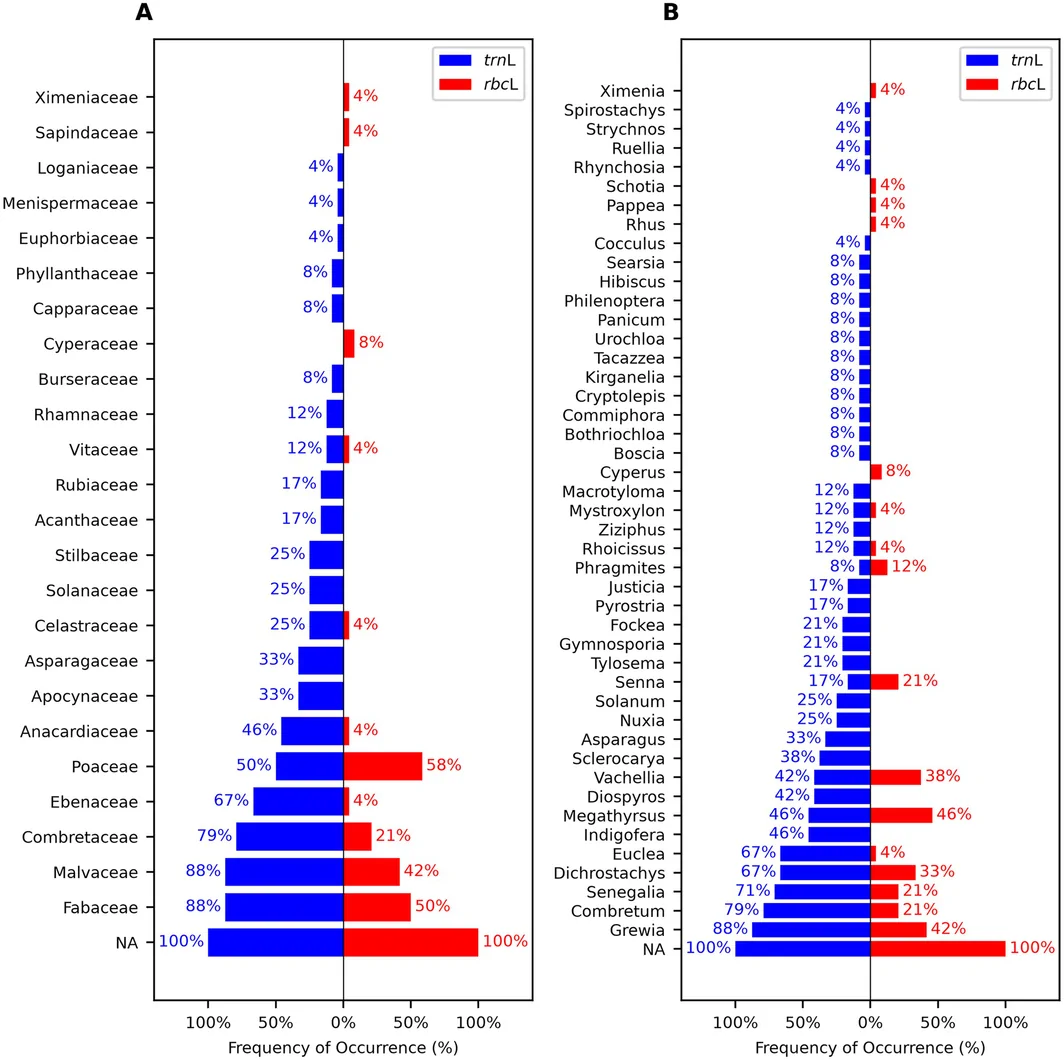
Quality‐control visualisation of frequency of occurrence (FOO%) for plant taxa detected in elephant faecal samples using the *rbc*L and *trn*L markers. Panels A and B show plant families and plant genera, respectively, as mirrored bar plots. Blue bars represent *trn*L detections and red bars represent *rbc*L detections. Unassigned taxa (‘NA’) and control columns were retained in this plot to show marker‐specific assignment success and sample recovery prior to final ecological filtering. Downstream dietary analyses were performed on cleaned diet matrices after removal of controls, contaminants and failed or unassigned‐only samples.

**FIGURE A6:**
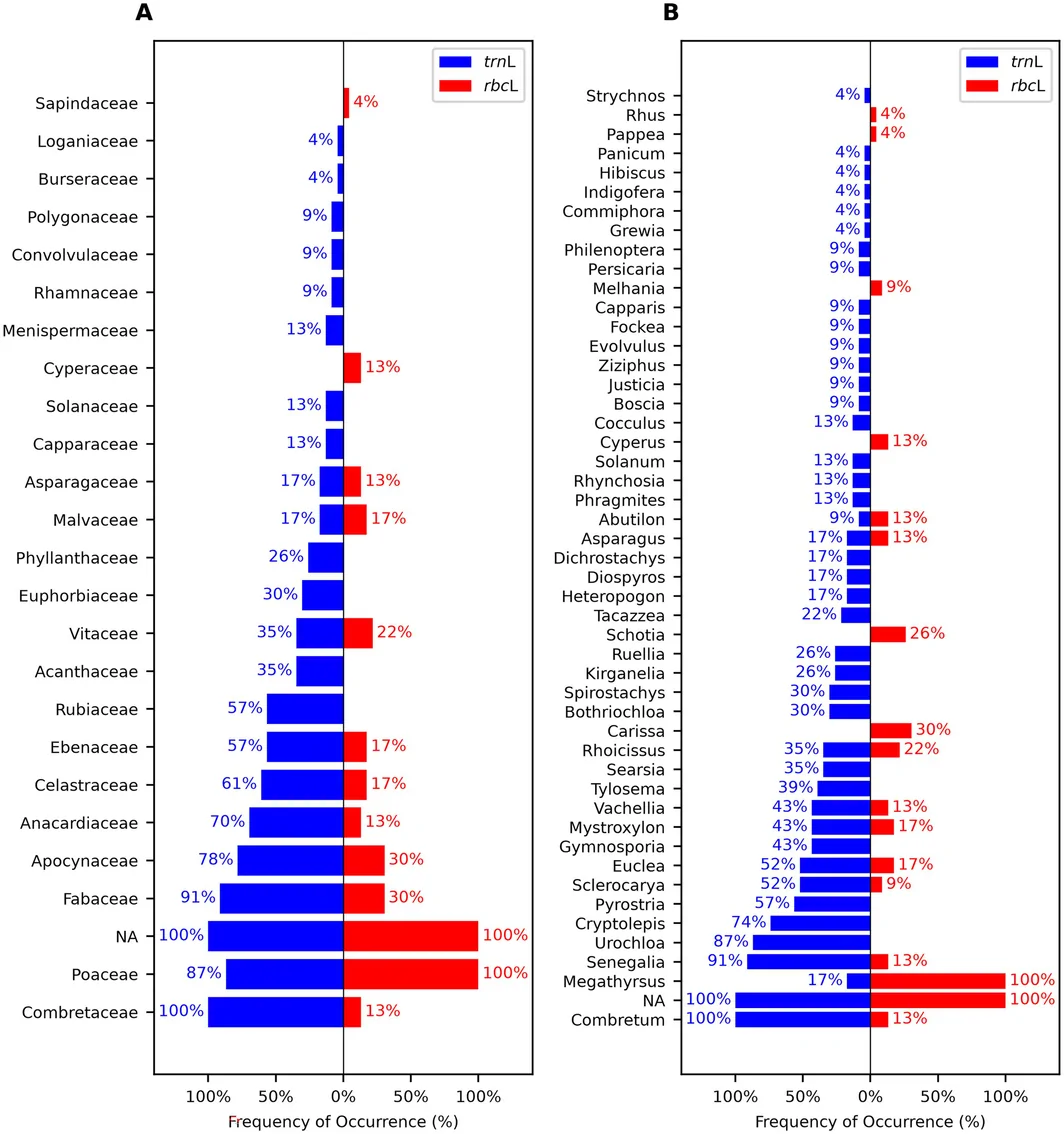
Quality‐control visualisation of frequency of occurrence (FOO%) for plant taxa detected in impala faecal samples using the *rbc*L and *trn*L markers. Panels A and B show plant families and plant genera, respectively, as mirrored bar plots. Blue bars represent *trn*L detections and red bars represent *rbc*L detections. Unassigned taxa (‘NA’) and control columns were retained in this plot to show marker‐specific assignment success and sample recovery prior to final ecological filtering. Downstream dietary analyses were performed on cleaned diet matrices after removal of controls, contaminants and failed or unassigned‐only samples.

**FIGURE A7:**
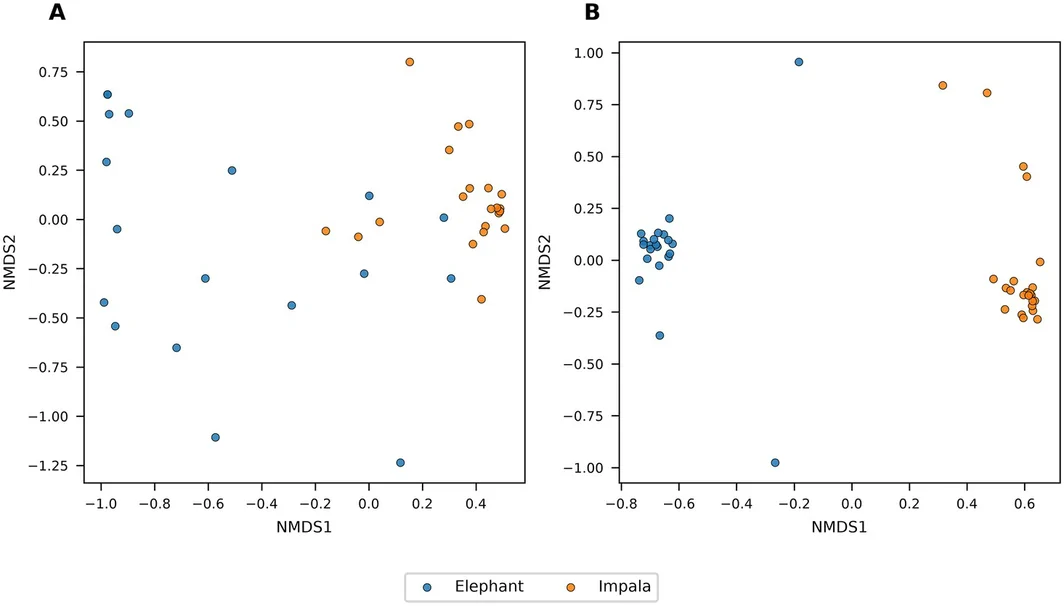
NMDS ordinations based on Bray‐Curtis dissimilarities of relative read abundance (RRA) data showing dietary composition of elephants and impalas for each DNA barcode marker. Panel A shows the *rbc*L dataset and panel B shows the *trn*L dataset. Points represent individual faecal samples and are coloured by consumer species. These ordinations provide an abundance‐weighted comparison to the primary FOO‐based Jaccard ordinations presented in the main text.

**FIGURE A8:**
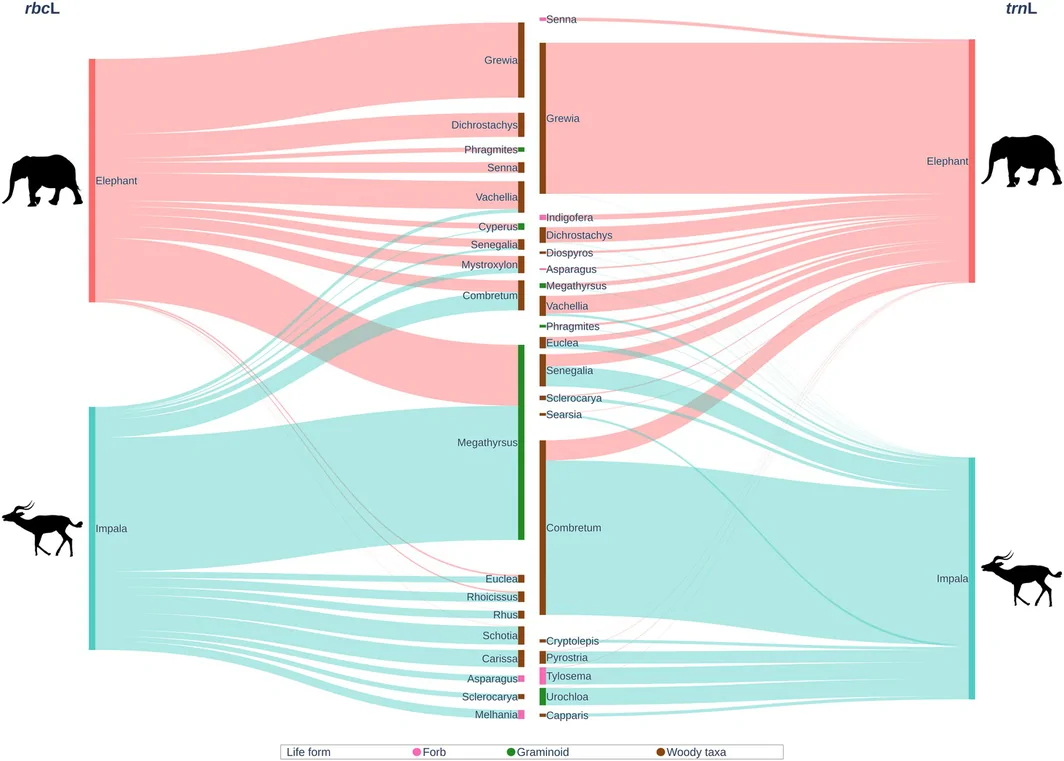
Relative read abundance (RRA)‐based Sankey diagrams showing dominant dietary genera of elephants and impalas for the *rbc*L and *trn*L datasets. Link width represents mean RRA, indicating each genus's proportional contribution to the diet. Only genera with mean RRA > 0.5% in at least one consumer were included. Plant genera are colour‐coded by life form: Woody taxa, graminoid, forb or succulent. Wider links show more dominant taxa and illustrate abundance‐weighted differences in dietary composition between consumers.

## Data Availability

Raw sequence data generated for this study have been deposited in NCBI under BioProject accession PRJNA1465205 and are available at: http://www.ncbi.nlm.nih.gov/bioproject/1465205.

## Appendix 1

This appendix provides additional dietary composition plots, quality‐control visualisations and relative read abundance (RRA)‐based analyses supporting the main frequency of occurrence (FOO%)‐based results. These include marker‐specific RRA summaries for elephant and impala samples, quality‐control FOO visualisations retaining unassigned taxa and control columns, RRA‐based NMDS ordinations, RRA‐based Sankey plots and a detailed taxonomic table of FOO% values used to support the genus‐level dietary interpretation.

**TABLE A1:** Sequences of the forward and reverse primers with their Illumina overhang adapters used for amplification of chloroplastic *rbc*L and *trn*L (UAA intron) barcodes from herbivore faecal samples in this study.

Barcode	Barcode sequence with Illumina overhang adapter (5′‐3′)	References
*rbc*L*a*‐F	TCGTCGGCAGCGTCAGATGTGTATAAGAGACA	Levin et al. (2003)
	GATGTCACCACAAACAGAGACTAAAGC	
*rbc*L*a*‐R	GTCTCGTGGGCTCGGAGATGTGTATAAGAGACAG	Kress and Erickson (2007)
	GTAAAATCAAGTCCACCRCG	
*trn*L*c*	TCGTCGGCAGCGTCAGATGTGTATAAGAGACAGC	Taberlet et al. (1991)
	GAAATCGGTAGACGCTACG	
*trn*L*d*	GTCTCGTGGGCTCGGAGATGTGTATAAGAGACAG	Taberlet et al. (1991)
	GGGGATAGAGGGACTTGAAC	

**TABLE A2:** Detailed taxonomic composition and frequency of occurrence (FOO%) of dietary items detected in elephant and impala faecal samples using *rbc*L and *trn*L DNA metabarcoding. The table includes family, genus, species, plant life form, elephant FOO%, impala FOO% and marker identity for each detected taxon.

Family	Genus	Species	Life form	Elephant FOO (%)	Impala FOO (%)	Marker
Malvaceae	*Abutilon*	*grandiflorum*	Forb	0	8.7	trnL
Asparagaceae	*Asparagus*	*falcatus*	Forb	38.1	17.4	trnL
Capparaceae	*Boscia*	*angustifolia*	Woody taxa	9.5	8.7	trnL
Poaceae	*Bothriochloa*	*insculpta*	Graminoid	9.5	30.4	trnL
Capparaceae	*Capparis*	*tomentosa*	Woody taxa	0	8.7	trnL
Menispermaceae	*Cocculus*	*hirsutus*	Woody taxa	4.8	13	trnL
Combretaceae	*Combretum*	*imberbe*	Woody taxa	90.5	100	trnL
Burseraceae	*Commiphora*	*africana*	Woody taxa	9.5	4.3	trnL
Apocynaceae	*Cryptolepis*	*oblongifolia*	Woody taxa	9.5	73.9	trnL
Fabaceae	*Dichrostachys*	*cinerea*	Woody taxa	76.2	17.4	trnL
Ebenaceae	*Diospyros*	*mespiliformis*	Woody taxa	47.6	17.4	trnL
Ebenaceae	*Euclea*	*divinorum*	Woody taxa	76.2	52.2	trnL
Convolvulaceae	*Evolvulus*	*alsinoides*	Forb	0	8.7	trnL
Apocynaceae	*Fockea*	*angustifolia*	Succulent	23.8	8.7	trnL
Malvaceae	*Grewia*	*bicolor*	Woody taxa	100	4.3	trnL
Celastraceae	*Gymnosporia*	*senegalensis*	Woody taxa	23.8	43.5	trnL
Poaceae	*Heteropogon*	*contortus*	Graminoid	0	17.4	trnL
Malvaceae	*Hibiscus*	*micranthus*	Forb	9.5	4.3	trnL
Fabaceae	*Indigofera*	*astragalina*	Woody taxa	52.4	4.3	trnL
Acanthaceae	*Justicia*	*flava*	Forb	19	8.7	trnL
Phyllanthaceae	*Kirganelia*	*reticulata*	Woody taxa	9.5	26.1	trnL
Fabaceae	*Macrotyloma*	*uniflorum*	Forb	14.3	0	trnL
Poaceae	*Megathyrsus*	*maximus*	Graminoid	52.4	17.4	trnL
Celastraceae	*Mystroxylon*	*aethiopicum*	Woody taxa	14.3	43.5	trnL
Stilbaceae	*Nuxia*	*oppositifolia*	Woody taxa	28.6	0	trnL
Poaceae	*Panicum*	*coloratum*	Graminoid	9.5	4.3	trnL
Polygonaceae	*Persicaria*	*decipiens*	Forb	0	8.7	trnL
Fabaceae	*Philenoptera*	*violacea*	Woody taxa	9.5	8.7	trnL
Poaceae	*Phragmites*	*australis*	Graminoid	9.5	13	trnL
Rubiaceae	*Pyrostria*	*hystrix*	Woody taxa	19	56.5	trnL
Vitaceae	*Rhoicissus*	*tridentata*	Woody taxa	14.3	34.8	trnL
Fabaceae	*Rhynchosia*	*minima*	Forb	4.8	13	trnL
Acanthaceae	*Ruellia*	*patula*	Forb	4.8	26.1	trnL
Anacardiaceae	*Sclerocarya*	*birrea*	Woody taxa	42.9	52.2	trnL
Anacardiaceae	*Searsia*	*pyroides*	Woody taxa	9.5	34.8	trnL
Fabaceae	*Senegalia*	*mellifera*	Woody taxa	81	91.3	trnL
Fabaceae	*Senna*	*occidentalis*	Forb	19	0	trnL
Solanaceae	*Solanum*	*incanum*	Woody taxa	28.6	13	trnL
Euphorbiaceae	*Spirostachys*	*africana*	Woody taxa	4.8	30.4	trnL
Loganiaceae	*Strychnos*	*madagascariensis*	Woody taxa	4.8	4.3	trnL
Apocynaceae	*Tacazzea*	*apiculata*	Woody taxa	9.5	21.7	trnL
Fabaceae	*Tylosema*	*fassoglense*	Forb	23.8	39.1	trnL
Poaceae	*Urochloa*	*mosambicensis*	Graminoid	9.5	87	trnL
Fabaceae	*Vachellia*	*xanthophloea*	Woody taxa	47.6	43.5	trnL
Rhamnaceae	*Ziziphus*	*mucronata*	Woody taxa	14.3	8.7	trnL
Malvaceae	*Abutilon*	*angulatum*	Forb	0	13	rbcL
Asparagaceae	*Asparagus*	*suaveolens*	Forb	0	13	rbcL
Apocynaceae	*Carissa*	*bispinosa*	Woody taxa	0	30.4	rbcL
Combretaceae	*Combretum*	*hereroense*	Woody taxa	23.8	13	rbcL
Cyperaceae	*Cyperus*	*compressus*	Graminoid	9.5	13	rbcL
Fabaceae	*Dichrostachys*	*cinerea*	Woody taxa	38.1	0	rbcL
Ebenaceae	*Euclea*	*divinorum*	Woody taxa	4.8	17.4	rbcL
Malvaceae	*Grewia*	*bicolor*	Woody taxa	47.6	0	rbcL
Poaceae	*Megathyrsus*	*maximus*	Graminoid	52.4	100	rbcL
Malvaceae	*Melhania*	*prostrata*	Forb	0	8.7	rbcL
Celastraceae	*Mystroxylon*	*aethiopicum*	Woody taxa	4.8	17.4	rbcL
Sapindaceae	*Pappea*	*capensis*	Woody taxa	4.8	4.3	rbcL
Poaceae	*Phragmites*	*mauritianus*	Graminoid	14.3	0	rbcL
Vitaceae	*Rhoicissus*	*revoilii*	Woody taxa	4.8	21.7	rbcL
Anacardiaceae	*Rhus*	*gueinzii*	Woody taxa	4.8	4.3	rbcL
Fabaceae	*Schotia*	*brachypetala*	Woody taxa	4.8	26.1	rbcL
Anacardiaceae	*Sclerocarya*	*birrea*	Woody taxa	0	8.7	rbcL
Fabaceae	*Senegalia*	*nigrescens*	Woody taxa	23.8	13	rbcL
Fabaceae	*Senna*	*bicapsularis*	Woody taxa	23.8	0	rbcL
Fabaceae	*Vachellia*	*schweinfurthii*	Woody taxa	42.9	13	rbcL
Ximeniaceae	*Ximenia*	*caffra*	Woody taxa	4.8	0	rbcL
